# Experimental and finite element analysis of a two wheeler handlebar subjected to semi active constrained layer damping treatment

**DOI:** 10.1038/s41598-025-14042-8

**Published:** 2025-08-08

**Authors:** Keerthan Krishna, G. T. Mahesha, Sriharsha Hegde, Satish Shenoy Baloor

**Affiliations:** Department of Aeronautical and Automobile Engineering, Manipal Institute of Technology, Manipal Academy of Higher Education, Manipal, Karnataka 576104 India

**Keywords:** Magnetorheological elastomer (MRE), Magnetic field, Mechanical vibrations, Handlebar, Rider’s comfort, Numerical simulation, Mechanical engineering, Computational methods

## Abstract

The study investigates the transient vibration analysis of two-wheeler handlebar and the optimization of vibration damping using constrained layer Magnetorheological Elastomers (MRE). The research explores how varying magnetic field strengths affect the damping properties of MRE, which is strategically implemented in the handlebar of a two-wheeler with a view to reducing vibrations and improving rider comfort. This study is a combination of numerical simulation and experimental data to highlight the effectiveness of semi-active damping systems under different magnetic fields. A sandwich beam was prepared at the laboratory for the damping material, and it was tested for the variation in damping ratio when the magnetic field was varied. The results demonstrate significant improvements in vibration attenuation when MRE damping ratios are optimized by varying the magnetic field, resulting in enhanced structural integrity and rider comfort.

## Introduction

Vibration analysis is a critical aspect of engineering, particularly in the context of two-wheelers such as motorcycles and scooters. These vehicles are frequently subjected to various vibratory forces during operation, leading to potential discomfort for the rider and accelerated wear and tear of the vehicle components^1,2^. One significant source of these vibrations is road irregularities. Uneven road surfaces induce vibrations that travel through the wheels, suspension system, and frame of the two-wheeler, affecting its stability and handling^3^. Minimizing the effect of these vibrations effectively requires an understanding of the sources and an implementation of strategic damping treatments^4^.

When a two-wheeler traverses an uneven or rough road, the irregularities in the surface cause sudden and unpredictable oscillations in the vehicle’s structure^5–8^. These vibrations can vary widely in frequency and amplitude, depending on the severity of the road conditions. High-frequency vibrations can lead to rider discomfort and fatigue, while low-frequency vibrations can impact the vehicle’s control and stability^9,10^. Over time, these continuous vibratory forces can cause mechanical components to loosen, wear out faster, or even fail prematurely.

Damping is a critical factor in mitigating the adverse effects of vibrations caused by road irregularities. Strategic damping treatments can significantly enhance the performance and comfort in two-wheelers. By focusing on specific strategic locations, such as the handlebar, seat mounts, and footrest, damping treatments can be targeted to areas where vibrations are most directly experienced by the rider. For example, the handlebar is a critical point of contact where vibrations can cause significant discomfort and reduce control. By incorporating damping materials or mechanisms at these points, the intensity of vibrations transmitted to the rider can be significantly reduced^11,12^.

Considering the analysis of these vibrations, transient analysis is a powerful simulation tool used to validate the effectiveness of damping treatments in two-wheelers. This type of analysis allows engineers to simulate the time-dependent response of the vehicle to dynamic loads, such as those caused by road irregularities. To effectively simulate the impact of damping treatments, it is essential to incorporate damping ratios into the transient analysis. The damping ratio is a dimensionless measure that indicates the extent of damping in the system relative to critical damping. A higher damping ratio implies more effective damping^13,14^, leading to quicker decay of vibrations. In ANSYS, various damping models such as Rayleigh damping, structural damping, and material-specific damping can be utilized. By assigning appropriate damping coefficients to different components and strategic locations in the model, the simulation can accurately reflect the real-world behavior of the vehicle. During the transient analysis, the equations of motion are solved over a specified period using numerical integration methods. This captures the time-dependent response of the system to the dynamic loads. Key output parameters, such as displacement, velocity, and acceleration at strategic locations like the handlebar and seat mounts, are monitored to evaluate the vibrational behavior^15,16^.

Vibration reduction or damping techniques are a critical aspect of engineering, impacting various fields such as mechanical systems, aerospace, and civil engineering. Among the various methods available, Magneto-Rheological Elastomers (MREs) have emerged as a promising solution due to their unique properties. This section compares MRE-based vibration reduction methods with traditional shock absorbers and alternative materials, highlighting their advantages and limitations. Table 1 shows the comparison of MRE based vibration absorbers with traditional absorbers and alternative materials giving an insight into the different features of MRE based damping materials.

**Table 1:** MRE vs traditional vibration reduction methods.

Feature	MRE-based absorbers	Traditional absorbers	Alternative materials/methods
Adaptability	Highly adaptable with tunable stiffness and damping properties^17,18^	Limited adaptability; fixed damping and stiffness characteristics	Moderate adaptability: some materials like EREs offer fast response times but require high voltages
Bandwidth	Broadband control with multiple natural frequencies^19^	Narrow bandwidth; effective only within specific frequency ranges	Limited bandwidth: some methods like active control offer broader range but are complex
Self-sensing	Built-in self-sensing capability reduces need for external sensors^17^	Requires external sensors for operation	Generally, require external sensors; few materials offer self-sensing capabilities
Power consumption	Low power consumption due to magnetic field control	Passive; no power consumption	High power consumption in active control methods
Complexity	Moderate complexity due to magnetic field generation and control systems	Low complexity; simple design and operation	High complexity in active control methods; moderate in ERE-based systems
Cost	Higher initial cost due to advanced materials and control systems	Lower cost; widely available and well-established technology	Moderate to high cost depending on the method
Maintenance	Lower maintenance due to self-sensing and adaptive features^17^	Higher maintenance due to fixed components and lack of adaptability	Moderate maintenance requirements: some methods require frequent calibration

In this paper, the numerical simulation of the effectiveness of semi-active damping system which is the Magnetorheological elastomer is carried out for one of the strategic locations i.e. Handlebar. Handlebar being a critical component of a two-wheeler in transferring vibrations from road to the rider, it is considered for the numerical simulation. Transient analysis of the handlebar for a two-wheeler vehicle was conducted to understand its dynamic response to real-world operating conditions, particularly focusing on vibrations. This analysis is essential to ensure the handlebar’s structural integrity and to enhance rider comfort by minimizing the adverse effects of vibrations transmitted through the handlebar during vehicle operation. The paper mainly discusses the methodology of carrying out the simulation and tests by assuming a smaller acceleration and displacements rather than real world values to understand the dynamic capabilities of MRE. The following sections provide the detailed design and methods carried out during the simulation.

## Materials and methods

In an actual vehicular test, an ICE two wheeler is converted into electric at the laboratory and on road testing was conducted^6^. Further, in this transient study of the test two-wheeler handlebar using ANSYS Workbench 2023 R2, structural steel is employed to simulate the material properties and behavior under dynamic loads. The process begins with defining the material properties of structural steel within the ANSYS Workbench 2023 R2 software, which includes specifying its density, Young’s modulus, and Poisson’s ratio. These properties are crucial for accurately simulating the mechanical response of the handlebar to applied forces. The properties of structural steel used for simulation are as shown in Table 2.

**Table 2:** Properties of magnetorheological elastomer.

Property	Symbol	Value	Units
Density	ρ	2700	kg/m^3^
Young’s modulus	E	1.02 × 10^6^	Pa
Poisson’s ratio	ν	0.2	
Damping ratio	ζ	0.0349/0.0389/0.0432	

Similarly, the damping material is placed at the handlebar mounts during the assembly^20^. The properties of the damping material are as shown in Table 2. Here, the damping material is modelled as a Hyper elastic model using Mooney-Rivlin model to incorporate the MRE sample at the handlebar mount. The Mooney-Rivlin model is a widely used constitutive model for describing the non-linear stress–strain behavior of elastomeric materials, such as rubber^21^. This model is particularly suitable for materials that exhibit large deformations and are incompressible or nearly incompressible. In ANSYS, the Mooney-Rivlin model can be employed to simulate the mechanical behavior of materials such as Magnetorheological Elastomers (MREs), which are used in various applications for their unique properties that change in response to a magnetic field^22,23^. In this study, the damping ratio is employed to show case the damping effect due to the placement of MRE based damping material.

### Mesh independence study

**Fig. 1:**
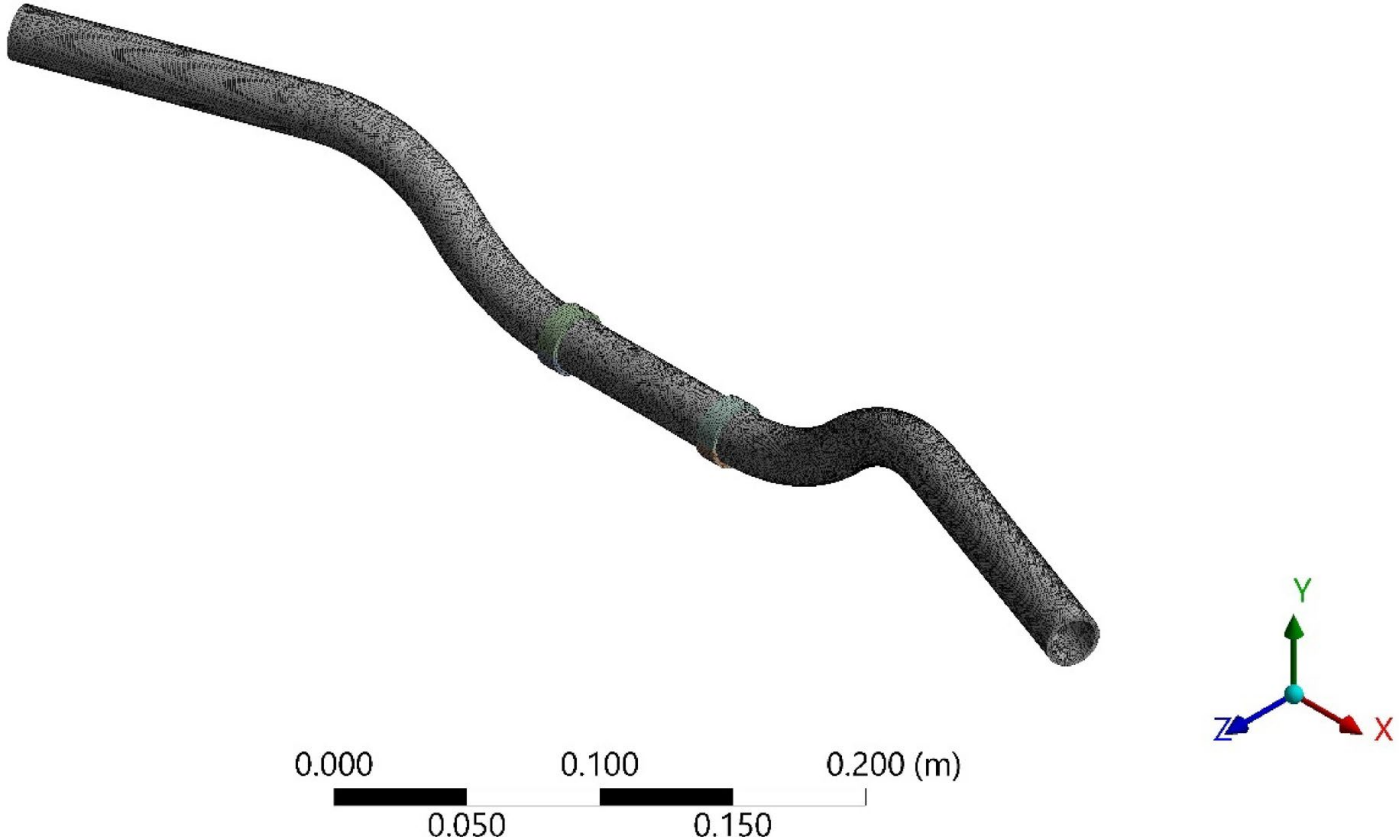
Meshed model of the handlebar with damping material.

After importing the 3D CAD model of the handlebar into ANSYS, the material assignment is carried out by selecting structural steel from the material library. This ensures that the software uses the correct mechanical properties during the simulation. The handlebar model is then meshed with tetrahedral elements, considering the complex geometry and critical areas that may experience higher stress concentrations. Several mesh sizes were developed by varying the mesh size from 4 to 1 mm. It was observed that mesh size less than 1 mm had no major changes in the results. Hence mesh size of 1 mm was used for all the analysis. The meshed model is shown in Fig. 1.

### Boundary conditions

**Fig. 2:**
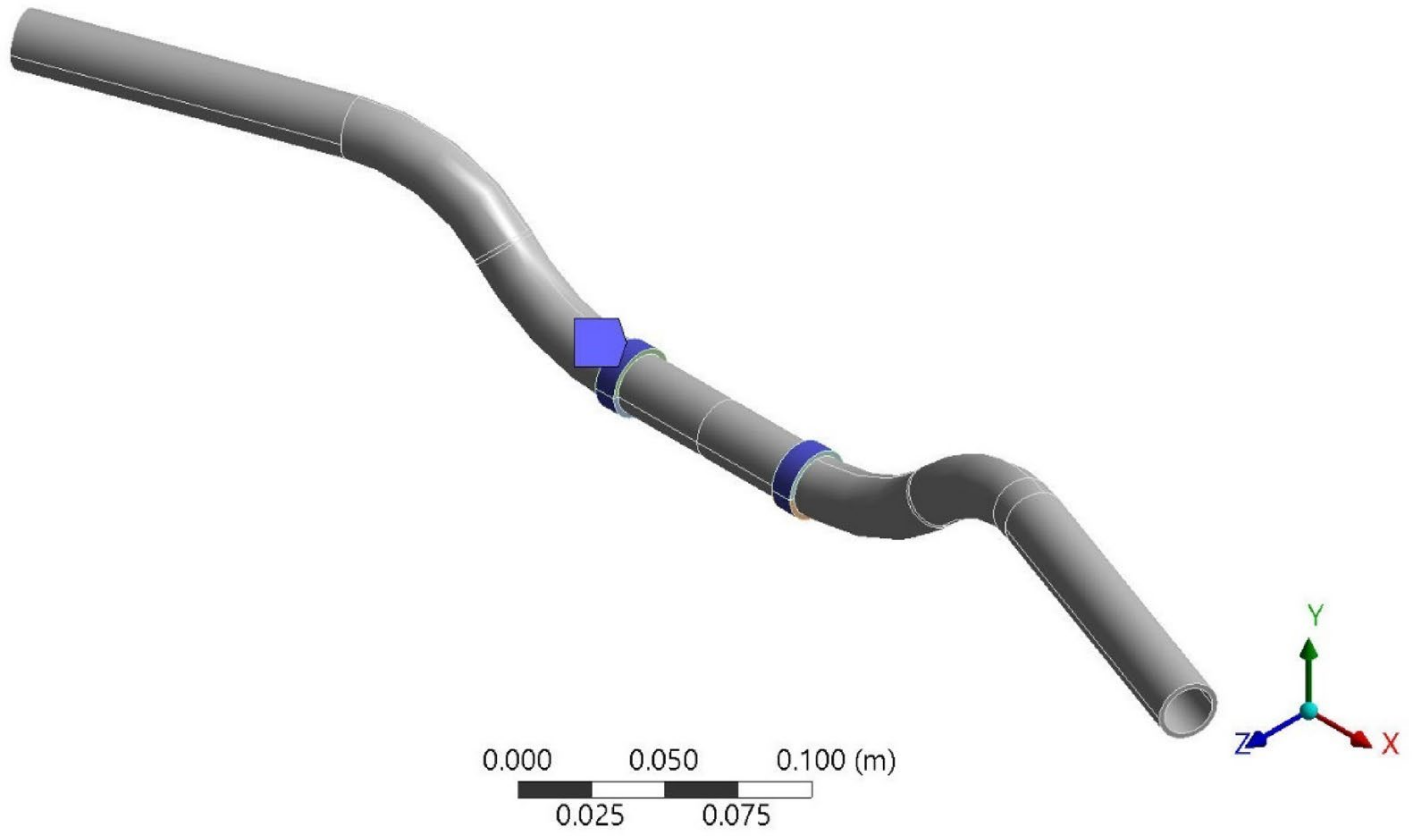
Boundary condition 1, fixed support (using Ansys workbench 2023 R2).

**Fig. 3:**
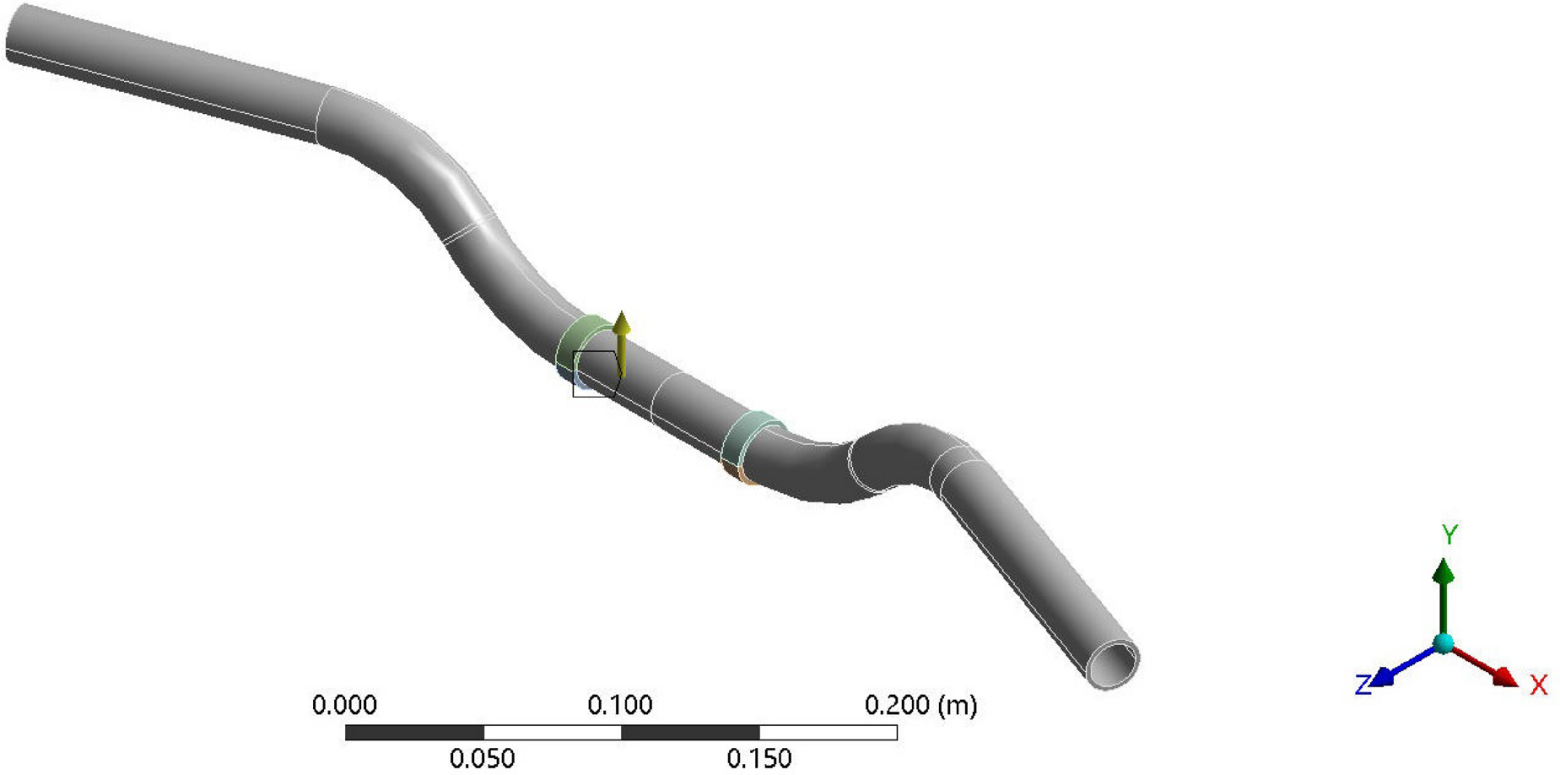
Boundary condition 2, acceleration (using Ansys workbench 2023 R2).

With the mesh ready, boundary conditions are applied. Fixed supports are defined at the mounting points where the handlebar fastens to the motorcycle frame. This mounting point is attached to the motorcycle frame in such a way where there are no relative movements involved. Hence this is fixed in all DOF. Figure 2 shows the fixed support of the handlebar. Transient loads, simulating real-world accelerations are applied to the handlebar. Hence, an acceleration of 1 m/s^2^ is applied on the handlebar. This acceleration is applied in order to simulate a real-world scenario where 1 m/s^2^ of acceleration was measured in the actual test^6^. Figure 3 shows the direction and magnitude of the applied acceleration.

### Variation of damping ratio of MRE at different magnetic field

To calculate the damping ratio of Magnetorheological Elastomer (MRE) under varying magnetic fields, the procedures are followed as per ASTM E756. This standard provides a comprehensive method for measuring the vibration-damping properties of materials, ensuring accurate and consistent results. The inclusion of magnetic fields in the test aimed to investigate their influence on the damping characteristics of MRE.

**Fig. 4:**
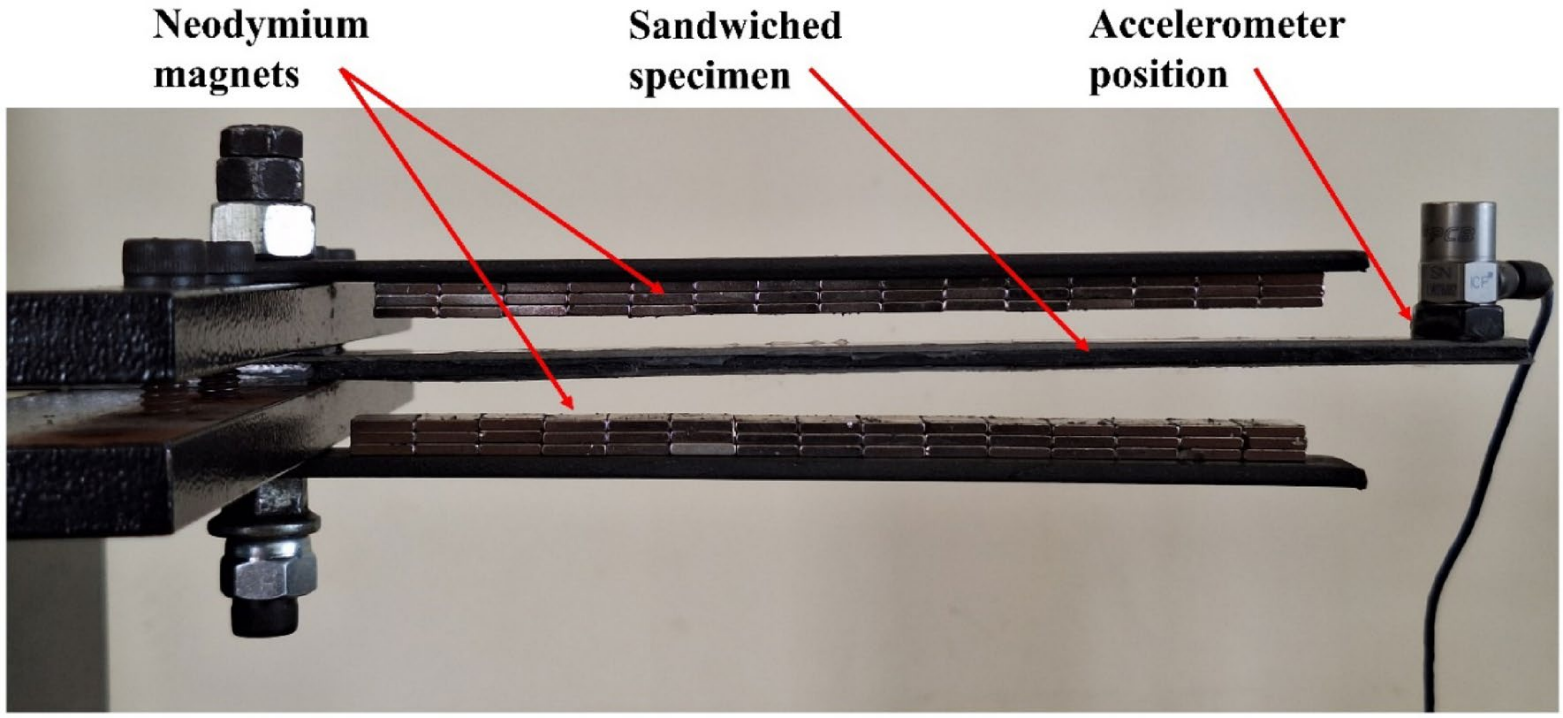
Experimental setup as per ASTM E756 standard.

The primary objective of this study was to determine how different intensities of magnetic fields affect the damping ratio of MRE. The damping ratio is a dimensionless measure that indicates how quickly a vibrating system dissipates its energy. By varying the magnetic field using neodymium magnets, the changes in the damping performance of MRE are observed in this study. The experimental setup followed the cantilever beam test configuration as prescribed by ASTM E756 as shown in Fig. 4.

The experimental setup for ASTM E756 involves a detailed configuration to measure the vibration-damping properties of a sandwiched Magnetorheological Elastomer (MRE) specimen. This setup begins with the preparation of the specimen, consisting of the MRE material sandwiched between two rigid layers, typically made of metal. In this study, stainless steel layers are used as they are non-magnetic and thus help in inducing magnetic field using permanent magnets. An MRE specimen with specified dimensions (180 mm × 10 mm × 2 mm) is prepared at the laboratory and is sandwiched between two stainless steel plates of the same dimensions. This sandwiched structure simulates practical applications where damping materials are often used in combination with rigid supports.

The prepared sandwiched MRE specimen is mounted as a cantilever beam in the test apparatus, with one end securely clamped to ensure it remains fixed, while the other end is free to vibrate. The free vibration test method is used to test the specimen and PCB Piezotronics made accelerometers are used to acquire the response. The accelerometers used (PCB Piezotronics) have a manufacturer-specified uncertainty of ± 1%. Triplicate tests showed a maximum standard deviation within ± 2%, ensuring measurement repeatability and accuracy. The accelerometers are connected to a data acquisition system that records the vibration data, allowing for precise measurement of the specimen’s response. During the test, the sandwiched MRE specimen is subjected to varying conditions, specifically different magnetic field strengths. The magnetic field is applied using neodymium magnets positioned around the specimen, and its intensity is carefully controlled and monitored. The data collected from the accelerometers is analyzed to determine the damping properties of the sandwiched MRE specimen. The analysis focuses on calculating key parameters such as the damping ratio, which indicates how effectively the specimen dissipates vibrational energy.

## Results and discussion

This section provides the detailed results and discussion of the obtained from the experimental investigation of the vibration-damping properties of the sandwiched Magnetorheological Elastomer (MRE) specimen under varying magnetic field strengths. The data collected through the ASTM E756 standard test method provided detailed insights into the damping behavior of the MRE material. By systematically varying the magnetic field intensity, it is aimed to understand its impact on the damping ratio and overall vibration response of the specimen. These damping ratios then used in the transient analysis of the handlebar provide the comparison of acceleration response at the handlebar proving the effectiveness of the semi-active damping system used in the experimentations.

### Analysis of effectiveness of semi-active damping

**Fig. 5:**
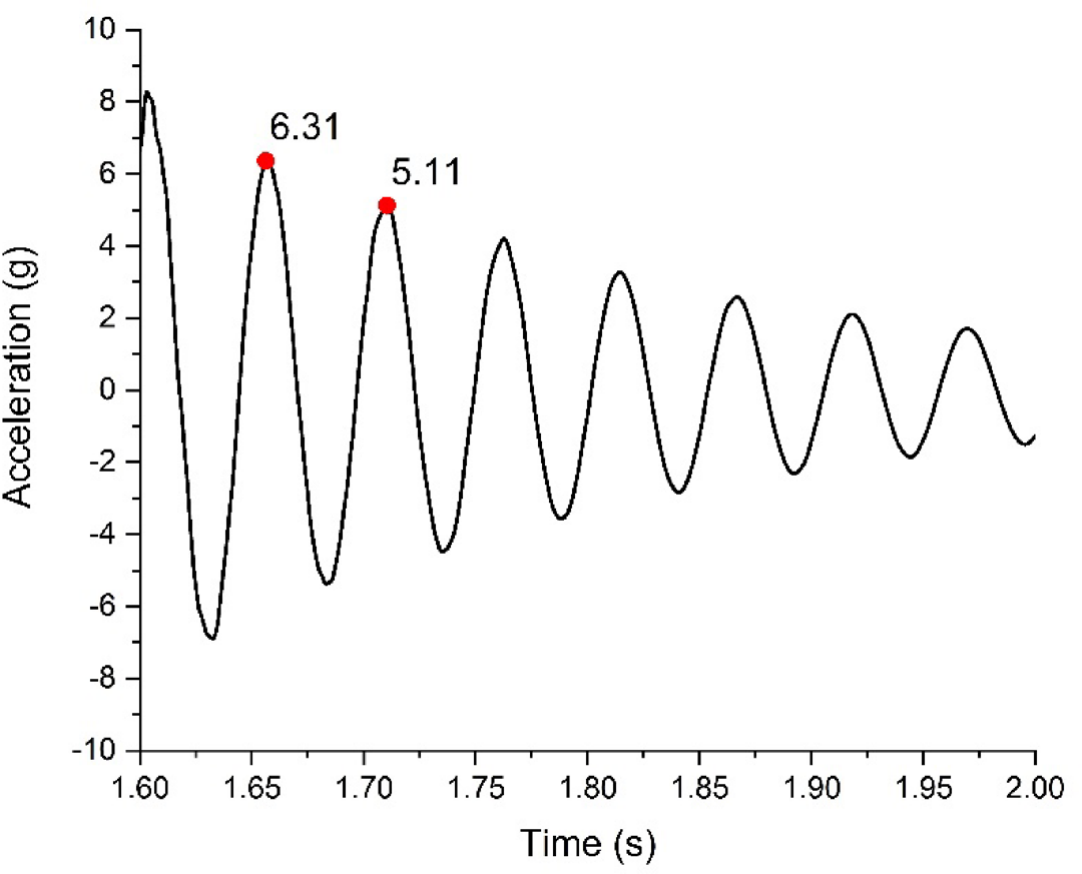
Calculation of damping ratio.

The analysis of effectiveness of the semi-active damping system carried out using ASTM E756 standard shows that as the magnetic field is varied the magnetorheological property of MRE is altered as well. From the experimentation as per ASTM E756 it can be observed that the damping properties of MRE increases with increase in the magnetic field. A typical graph of calculation of damping ratio is shown in Fig. 5.

1 $$\delta = ln \frac{x1}{{x2}}$$

2 $$\delta = \frac{2\pi \zeta }{{\sqrt {\left( {1 - \zeta^{2} } \right)} }}$$

In Eq. (1), Logarithmic decrement (δ) is a measure of the rate at which oscillations decrease in an underdamped system. It is defined as the natural logarithm of the ratio of successive amplitudes in free vibration. The damping ratio is then calculated using the logarithmic decrement. The relationship between the logarithmic decrement and the damping ratio is given in Eq. (2). This damping ratio is then adopted in the properties of the damping material in the simulation. Table 3 shows the variation of damping properties obtained at different magnetic fields.

**Table 3:** Damping properties at different magnetic field.

Magnetic field	Damping ratio	Elastic modulus (MPa)
4 mT	0.0349	23.99
8 mT	0.0389	19.92
12 mT	0.0432	7.71

**Fig. 6:**
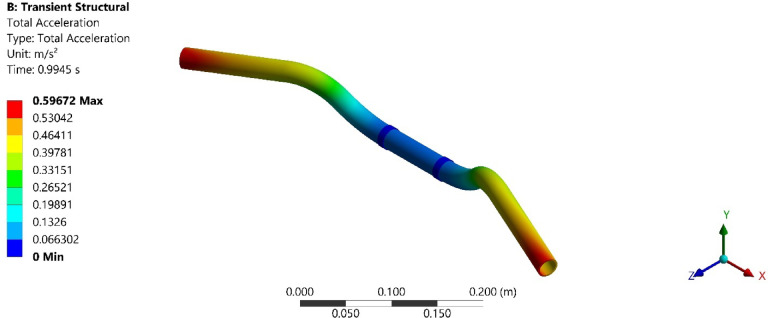
Total acceleration at damping ratio of 0.0349.

**Fig. 7:**
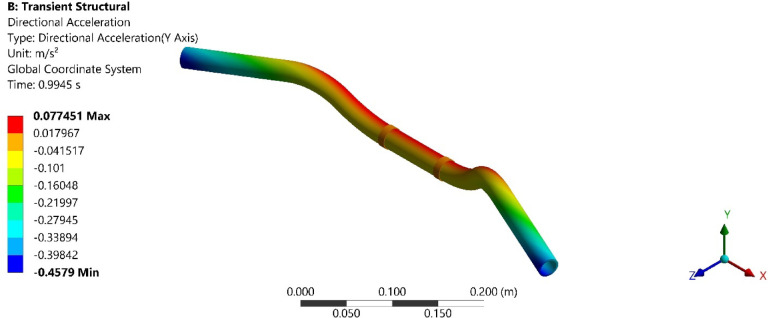
Directional acceleration at damping ratio of 0.0349.

**Fig. 8:**
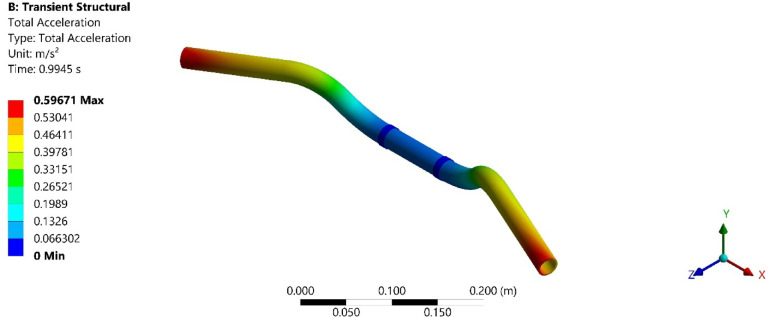
Total acceleration at damping ratio of 0.0389.

**Fig. 9:**
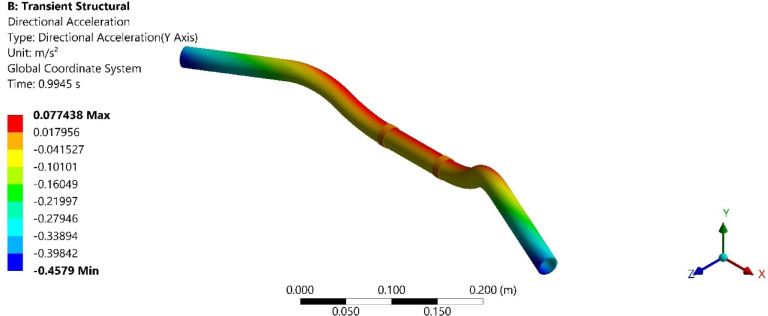
Directional acceleration at damping ratio of 0.0389 s.

**Fig. 10:**
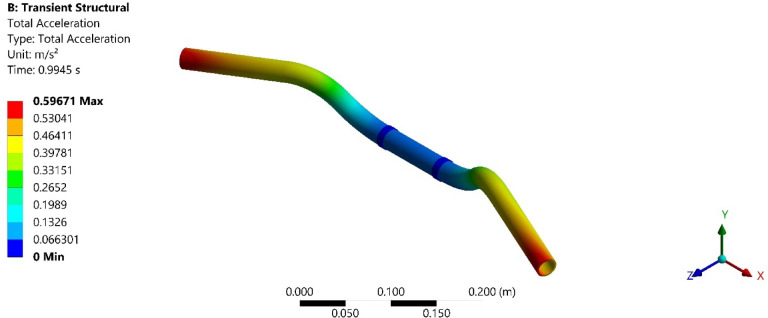
Total acceleration at damping ratio of 0.0432.

**Fig. 11:**
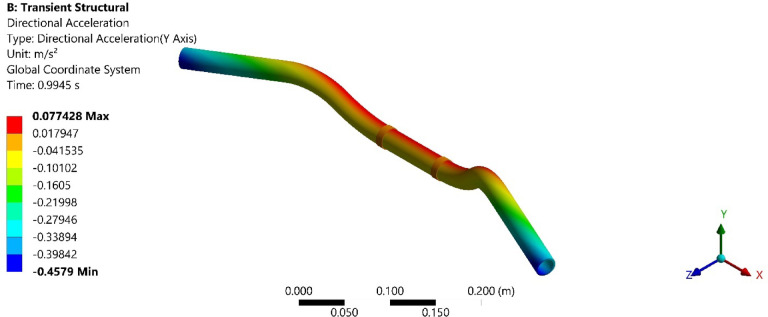
Directional acceleration at damping ratio of 0.0432.

On conducting the transient analysis of the handlebar, the acceleration response was found at the handlebar grip. In ANSYS, total acceleration and directional acceleration are crucial for understanding the dynamic behavior of structures under various loading conditions. Total acceleration refers to the vector sum of all the accelerations acting on a point in a structure. Directional acceleration, on the other hand, refers to the acceleration component along a specific direction, typically along one of the principal axes (x, y, or z). This helps in understanding how the structure responds to dynamic loads in specific directions. The total and directional accelerations of the handlebar after transient analysis are shown in Figs. 6, 7, 8, 9, 10 and 11. Where Fig. 6, shows the total acceleration of the handlebar by incorporating the damping ratio of 0.0349. Figure 7 shows the directional acceleration of the handlebar by incorporating the damping ratio of 0.0349 in the y-direction. Observing the directional acceleration of the handlebar in y-direction the maximum acceleration response of 0.077451 m/s^2^ is noted. This shows that the handlebar is lightly damped under the magnetic field of 0.4 mT. Hence, for better reduction of vibrations, a higher damping ratio needs to be incorporated. Hence, in the case of MRE, higher magnetic field leads to better damping of vibrations.

Furthermore, Fig. 8, shows the total acceleration of the handlebar by incorporating the damping ratio of 0.0389 and Fig. 9, shows directional acceleration. Here, it is noted that the y-directional acceleration has decreased by 0.016% when the magnetic field was increased to 0.8 mT and the corresponding damping ratio of 0.0389. The reduction in y-directional acceleration also highlights the importance of fine-tuning the magnetic field to optimize damping performance. Even a slight adjustment in the magnetic field strength led to noticeable improvements in vibration reduction, contributing to a smoother and more controlled riding experience. The results underscore the potential of using MREs with adjustable magnetic fields to achieve targeted vibration control.

Conducting the transient analysis for another step under the magnetic field of 1.2 mT and damping ratio of 0.0432, it is observed that the acceleration in the y-direction has further decreased by 0.029%, and this proves to be a better vibration damping. This study shows that as the magnetic field was increased at the MRE, the damping ratio of MRE enhances and thereby increases the rider’s comfort. Figure 10, shows the total acceleration of the handlebar at the damping ratio of 0.0389 and Fig. 11, shows directional acceleration.

**Fig. 12:**
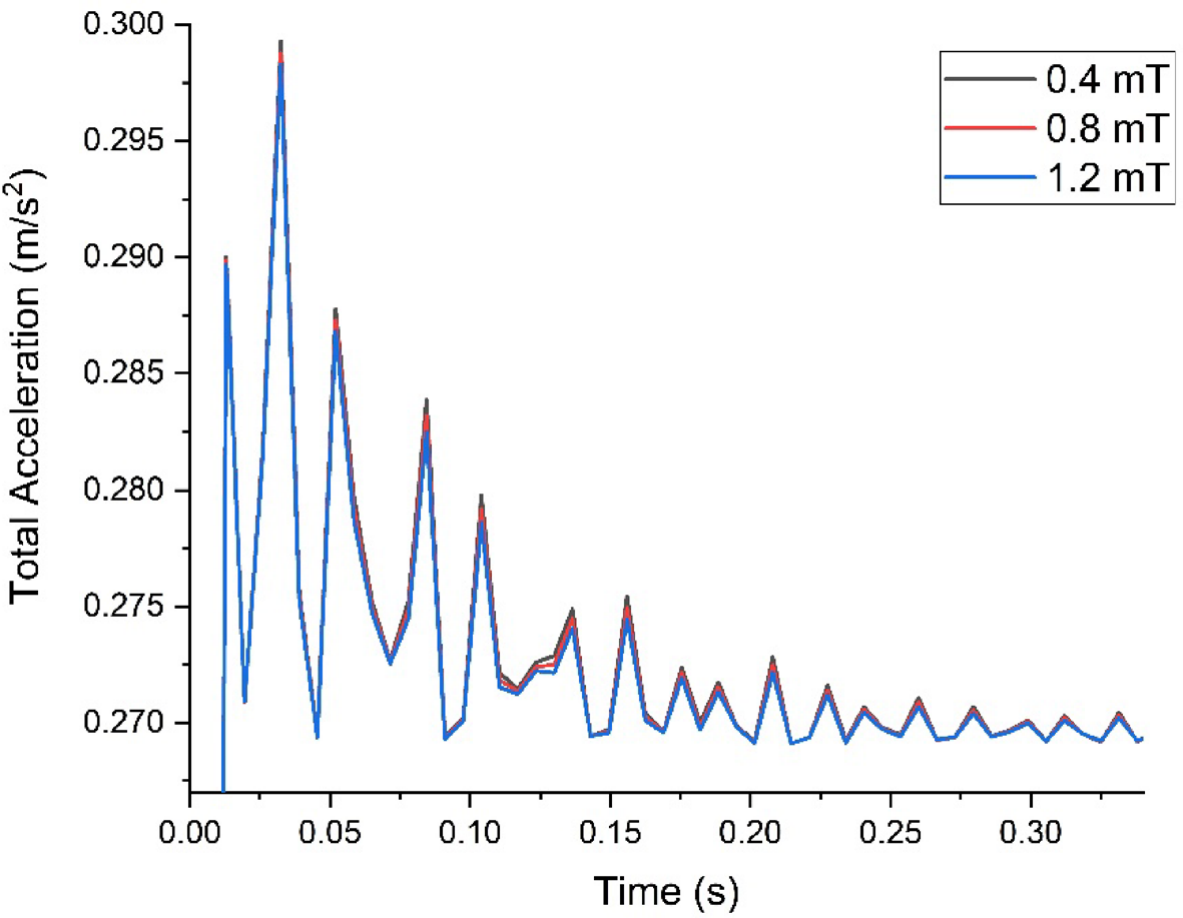
Comparison of total acceleration.

**Fig. 13:**
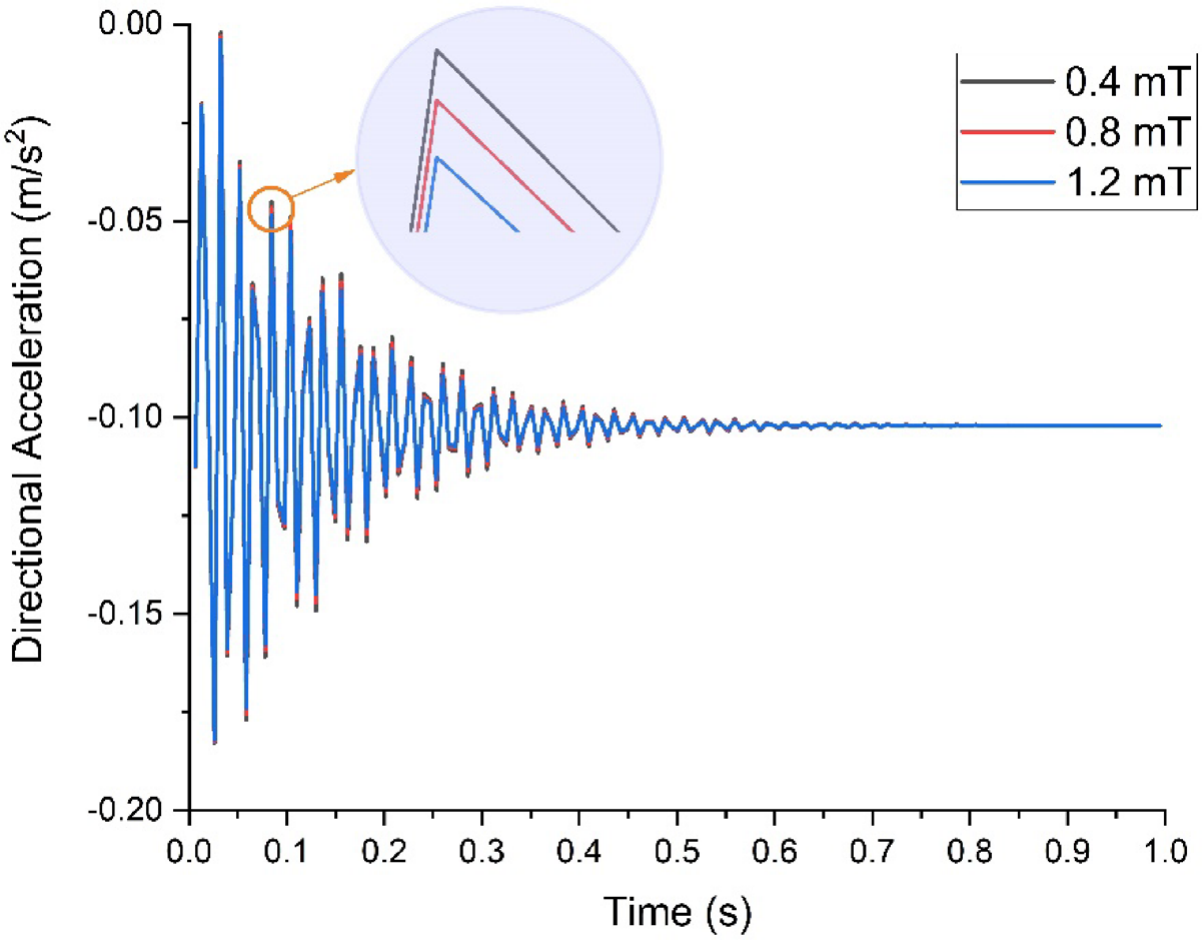
Comparison of Directional acceleration.

Through this analysis, a decrease in both total acceleration and directional accelerations after damping treatment was observed. This indicates that the damping measures are effectively reducing vibrations, leading to improved comfort, enhanced structural integrity, increased safety, and optimized performance of the system. The overall decrease in total and directional accelerations with damping treatments is shown in Figs. 12 and 13 respectively. With the application of a 1.2 mT magnetic field using permanent magnets, the maximum possible damping is achieved at the handlebar ensuring improved damping properties.

## Conclusion

This research investigates the effectiveness of semi-active damping system at the strategic location of the electric two-wheeler. Based on the calculation of damping ratios as per ASTM E756 standard for the MRE specimen by varying the magnetic field, these inputs are fed into the simulation of transient analysis of the handlebar. Based on the method of damping at the handlebar and its acceleration response, the following conclusions are drawn:

- The damping properties of MRE was increased by 11.46% when the magnetic field was increased from 4 mT to 8 m, and it was further increased by 23.78% at the magnetic field of 12 mT.
- The y-directional acceleration has decreased by 0.016% when magnetic field is increased to 8 mT with damping ratio of 0.0389.
- The y-direction had further decreased by 0.029% when magnetic field was increased to 12 mT with damping ratio of 0.0432.
- The changes in magnetic field in turn altered the properties of MRE and improved the rider comfort. This shows the effectiveness of semi-active damping system.

## Discussion

Our previous paper^6^ focused on experimentally evaluating the effectiveness of magnetorheological elastomer (MRE) and silicone rubber damping treatments on an electric two-wheeler under actual road conditions. It involved comprehensive on-road tests conducted at different speeds, capturing the real vibration environment experienced by the vehicle. The study demonstrated significant reductions in weighted RMS acceleration and vibration dose value, particularly when MRE with magnetic field was used, thereby proving its practical capability to enhance rider comfort.

However, the current paper is a proof-of-concept simulation study aimed at understanding the behavior of MRE damping in a controlled numerical environment. Unlike the experimental study, it did not employ real road vibration inputs but instead used a simplified acceleration input to observe the effect of varying damping ratios on the handlebar’s transient response. While the simulation showed only small percentage reductions in vibration levels, it effectively demonstrated the conceptual feasibility of implementing MRE in a two-wheeler handlebar model. Thus, while the experimental paper provided practical validation under real riding conditions, the current simulation paper established a theoretical foundation for modelling MRE damping, paving the way for future studies where real-world experimental inputs can be incorporated into simulations to achieve direct quantitative validation. Overall, both studies complement each other, with the experimental work proving practical applicability and the simulation work strengthening the theoretical understanding and design optimization potential for semi-active damping systems.

### Future scope

This study mainly focusses on the methodology and proof of concept for the simulation of MRE damping material for better comfort in two wheelers. Even though the percentage of reduction in the current study is not very sufficient to conclude the significance of MRE, upon the application of real-world test data using proper standards will give significant reductions in the vibration values. In this regard, the future scope of the study is mainly as indicated below:

- Incorporation of real-world data of vibrations to the simulation and thereby get the actual vibration reduction percentages.
- Modelling and simulation of the entire vehicle to get the accurate acceleration values and thereby observe the effect of damping treatment.
- Rider perception studies and subjective feedback measurements could allow the damping to be completely semi-active damping.
- While this study varied damping ratios to observe acceleration response trends, a detailed parametric sensitivity analysis (e.g., variation of loading, material properties, boundary conditions) is a future study to comprehensively evaluate the robustness of simulation predictions.

## Data Availability

The authors declare that the data supporting the findings of this study are available within the article. The generated datasets are also available from the first [K.K] and the corresponding authors [S.S.B] on reasonable request. All experiments are conducted as per the guidelines and regulations in ISO 2631-1 and ASTM E756 standards. An Indian patent has been filed with application number “202,441,030,281” titled “AN APPARATUS FOR DAMPING VIBRATIONS FOR COMFORTABLE RIDING ON A TWO-WHEELER VEHICLE” and is published in the patent office journal on 19/04/2024^17^.
